# What is New and Innovative in Emergency Neurosurgery? Emerging Diagnostic Technologies Provide Better Care and Influence Outcome: A Specialist Review

**DOI:** 10.1155/2013/568960

**Published:** 2013-11-14

**Authors:** Athanasios K. Zisakis, Vassilios Varsos, Aristomenis Exadaktylos

**Affiliations:** ^1^Department of Neurosurgery, Red Cross Hospital, 1st Erythrou Staurou and Athanasaki Street, 11526 Ampelokipoi, Athens, Greece; ^2^Department of Emergency Medicine, Inselspital, 3010 Bern, Switzerland

## Abstract

The development of emergency medical services and especially neurosurgical emergencies during recent decades has necessitated the development of novel tools. Although the gadgets that the neurosurgeon uses today in emergencies give him important help in diagnosis and treatment, we still need new technology, which has rapidly developed. This review presents the latest diagnostic tools, which offer precious help in everyday emergency neurosurgery practice. New ultrasound devices make the diagnosis of haematomas easier. In stroke, the introduction of noninvasive new gadgets aims to provide better treatment to the patient. Finally, the entire development of computed tomography and progress in radiology have resulted in innovative CT scans and angiographic devices that advance the diagnosis, treatment, and outcome of the patent. The pressure on physicians to be quick and effective and to avoid any misjudgement of the patient has been transferred to the technology, with the emphasis on developing new systems that will provide our patients with a better outcome and quality of life.

## 1. Introduction

When an emergency care system matures, the scope of emergency medicine (EM) expands. Most emergency department care is provided by specially trained physicians. There is a mature academic professional organization that can advance the field, which can now sustain fellowships and subspecialization, national databases, and peer reviewed journals. Emergency medical services systems are developed and run on city, regional, and even national levels. Internationally, two different systems have been developed for the delivery of emergency medical care. The first is the Anglo-American model that is familiar within the United States and the other is the Franco-German model. The Anglo-American model provides prehospital care. Paramedic and emergency medical technicians extend the role of the physician, caring for, stabilizing, and transporting the patient. On the other hand, in the Franco-German model, care is brought to the patient. Emergency systems used to provide out-of-hospital urgent care and to screen the patients to decide who needs to be transferred. The physician and the technology are sent to the scene in the hope of providing an immediate high level of care when most needed. Management systems are currently in place for process improvement, quality assurance, and cost controls. Countries that can be described as having mature emergency care systems include Australia, Canada, the United Kingdom, Germany, Switzerland, and the United States [1].

## 2. The Necessity of New Technology: Telemedicine

Rapid assessment of injuries and life-preserving therapy is required, but defining the optimal strategy can be complicated when multiple organ systems are involved [2]. The improvements needed in emergency neurosurgery can be supported by introducing new tools for specialists.

Although the gadgets and tools that the neurosurgeon uses today in emergencies give him important help in diagnosing and treating the patient, we still need new technology. This should accelerate diagnosis and treatment. Moreover, it must be user-friendly, so that it can be used even by a junior physician and still give excellent results. Furthermore, modern technology concentrates on avoiding misdiagnosis, as this can lead to disastrous results for the patient. Additionally, rapid and specific diagnosis can always help to improve the patient's quality of life.

Another very important instrument that has to be developed for neurosurgical emergencies is the telemedical emergency neurosurgical network. Teleeducation, teleconferencing, and teleconsultation have flourished, albeit mainly as a showcase between a number of emergency units within leading institutes [3, 4].

With advances in information and imaging technology, the application of robotic systems to the health sector has become a burgeoning field in assisting surgeons in manipulating, monitoring, and/or guiding operations, with the advantages of high targeting precision, reliable support during protracted operations, and the possibility of preoperative planning based on patients' images. In 2001, around 270,000 image-guided robotic operations were conducted annually worldwide [3, 4].

In the developing countries, telemedicine holds promises increasingly balanced access to health care services. To this end, collaboration with the developed countries will increase and will help to ensure that expertise, knowledge, and experience are more rapidly and more cheaply available [3].

## 3. Novel Neurosurgical Tools

There have recently been rapid developments in medical technology in emergency departments. The present paper presents a spectrum of neurosurgical tools that have been developed in the last two years.

**Figure 1:**
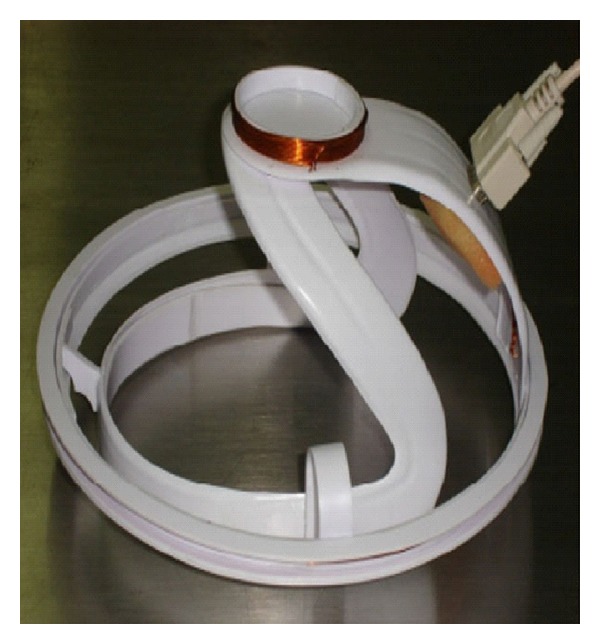
Brain injury detector based on Volumetric Electromagnetic Phase Shift Spectroscopy. Courtesy of UC Berkeley.

Researchers at UC Berkeley have been working on developing a new brain injury detector that is cheap, easy to use, and can provide nearly immediate results. This device sends radio signals that pass through the brain and are detected using a special antenna. The underlying technology is known as “Volumetric Electromagnetic Phase Shift Spectroscopy (VEPS)” and can detect changes in tissue properties inside the body through noncontacting, multifrequency electromagnetic measurements from the exterior of the body, thus providing rapid and inexpensive diagnostic testing (Figure 1) [5, 6]. This is a diagnostic method for brain injury that can be used in poor rural areas where the population has no access to advanced medical technology and services [5]. Thus, the patient will receive a rapid diagnosis, even from nonspecialized personnel, and it can then be decided whether to transfer him to advanced medical services for further neuromonitoring, which can be crucial for the outcome and the quality of life of the patient. Only time will tell which of these technological solutions will become commonly used in the future to help diagnose brain trauma, and the VEPS headset is only completing small trials at the moment, so it is still far from reaching the rural communities it hopes to help. Still, the innovation in this field shows that we may have improved treatments and diagnosis for traumatic brain injury not only rapidly but also well ahead of time [7, 8].

**Figure 2:**
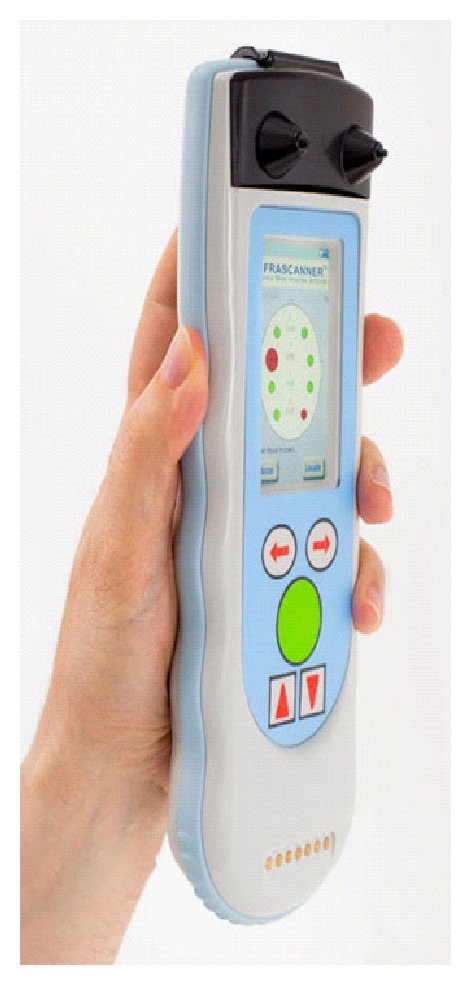
The infrascanner intracranial haematoma detector, model 2000. Courtesy of Infrascan Handheld brain diagnostics.

The updated infrascanner model 2000 intracranial haematoma detector is another tool for the neurosurgeon that has received FDA approval (Figure 2). This is a device for detecting intracranial haematomas. It is meant to be a simple, easy-to-use screening tool, which can be used to identify high risk patients requiring further workup, including CT [9]. An estimated, 1.5 million individuals seek medical treatment for head trauma in the USA each year, and a total of 10 million individuals seek head trauma treatment annually worldwide [10–15]. Intracranial haematomas resulting from a traumatic brain injury are life-threatening and have been reported to occur as the primary injury in 40% of patients with severe head injury [10–15]. Successful treatment often relies upon timely diagnosis and intervention prior to neurological deterioration. The early identification of a brain haematoma can play a significant role in facilitating transportation of critically injured patients to facilities which can verify Infrascanner's early diagnosis and offer surgical intervention. “Before the Infrascanner, first responders had to rely on imprecise methods to detect brain bleeds in patients, potentially delaying treatment,” said Dr. Joseph Maroon, Professor and Vice Chairman of the Department of Neurological Surgery at the University of Pittsburgh, team neurosurgeon for the Pittsburgh Steelers, and Medical Advisory Board member of civilian Infrascanner distributor, MedLogic, LLC. “Whether on the field of battle with military medical personnel or on the thousands of playing fields with sports health professionals in professional, amateur, or youth sports, the Infrascanner can potentially save lives by quickly detecting life-threatening brain bleeds earlier,” added Dr. Maroon. It seems to be the frontier for the further management of a potential patient with haematoma. This instrument can be easily used by any physician and can immediately demonstrate whether a haematoma is present or not. The first physician at the scene of injury can then decide whether the patient should be immediately transferred to an advanced medical centre to be treated and monitored. There is always the possibility of false positive results, which can be considered as minor faults, since the haematoma patients will be diagnosed immediately and the acute transfer to a neurosurgical department would be beneficial for the patient. This device uses near-infrared technology to detect intracranial bleeding. Extravascular blood absorbs near-infrared light more than intravascular blood, due to the higher concentrations of haemoglobin in an acute haematoma compared to brain tissue. The scanner measures the difference in near-infrared absorption and provides a simple signal that indicates whether bleeding is likely to be present [9, 16].

**Figure 3:**
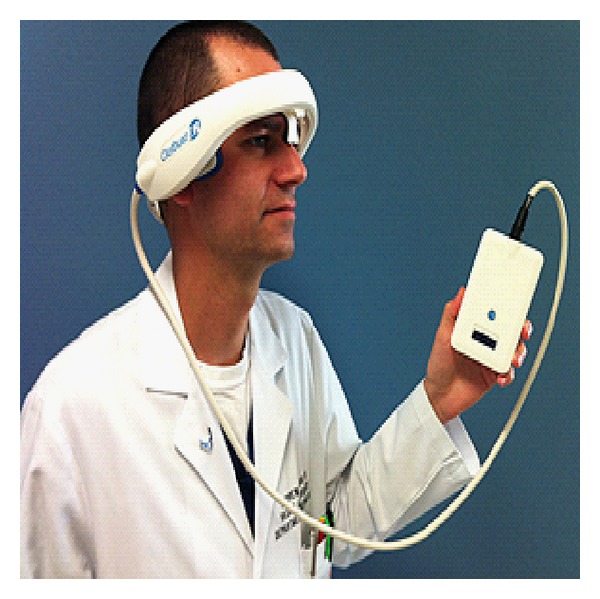
The Clotbust ER ultrasound system for thrombolysis. Courtesy of Cerevest Therapeutics Inc.

The Clotbust ER is another important ultrasound system that has received the CE mark and that is potentially an important tool for the emergency department (Figure 3). This is an ultrasound system from Cerevest Therapeutics, Inc., and is a sonolysis system used to treat ischaemic stroke in emergency settings. The Clotbust ER is designed to deliver therapeutic ultrasound energy noninvasively to occluded blood vessels in the brain, together with standard intravenous thrombolytic therapy. The energy of the ultrasound beam is transformed into energy of fluid motion. At very low pressures, this streaming inside the brain causes mild stirring, leading to the exposure of additional fibrin sites to plasmin. Integrated software controls the delivery of consistent therapeutic levels of energy required to attain acoustic streaming, which makes the device operator-independent and it avoids the need for an experienced ultrasound specialist [17–19]. Some clinical studies have suggested that when ultrasound energy is applied during conventional intravenous tPA thrombolysis, the process of clot lysis is augmented, allowing more rapid resolution of blood flow to the ischaemic brain. This process is termed sonothrombolysis. The hope is that in the future, IV thrombolysis may be superseded by the use of cavitating microspheres in conjunction with sonothrombolysis, which may hopefully enhance clot dissolution further. The small size of these spheres (1.2 microns) allows them to penetrate and pass through the fibrin matrix of the blood clot [20, 21].

This seems to be an essential tool for the physician in the emergency department. With this tool, the physician (even when he is not a specialist) can noninvasively deliver ultrasound energy to a patient who has just been diagnosed with an ischaemic stroke in the ER. The thrombus starts to dissolve and can be then treated with a standard intravenous thrombolytic agent. That is extremely important, since this can provide a better outcome and better quality of life for the patient with ischemic stroke, without the need for specialised personnel. 

**Figure 4:**
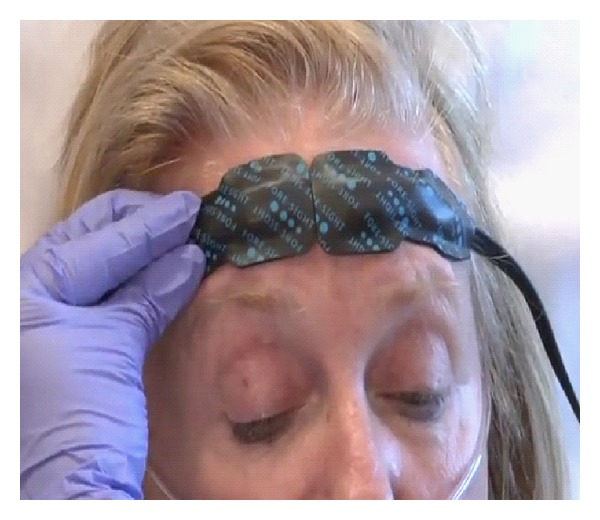
The Fore-sight monitor for cerebral blood flow in stroke treatment. Courtesy of Casmed, Brandord, CT, USA.

For stroke treatment and monitoring in the emergency department, attention must be drawn to the new device called Fore-sight from Casmed of Branford, CT, USA (Figure 4) [22]. A clinical evaluation led by Dr. David MacLeod at Duke University Medical Center in Durham, NC, USA, examined the relationship of cerebral tissue oxygen saturation (SctO2) measured by fore-sight to longer term postoperative cognitive decline 6 weeks after surgery. New clinical study results show that casmed's fore-sight technology provides superior accuracy and potential for improved outcomes. Decreased forebrain cerebral tissue oxygen saturation is associated with cognitive decline after cardiac surgery [22]. Subjects were given a battery of cognitive tests, both before and 6 weeks after surgery, for verbal memory and language comprehension, figure memory, attention and concentration, and psychomotor and processing speed. This observational study showed that decreased intraoperative SctO2 levels are potentially associated with longer term forebrain postoperative cognitive decline. It is believed that this was the first study to provide initial evidence that longer term postoperative cognitive decline may be associated with decreased cerebral tissue oxygen saturation measured by cerebral oximetry [23]. This device measures blood oxygen in a similar manner to a finger clip pulse oximeter. Continuous near-infrared regional cerebral perfusion monitoring is provided in stroke patients. The sensors stick on like adhesive bandages above each of the patient's eyebrows and emit near-infrared light that penetrates the scalp and underlying brain tissue [24]. As with Clotbust, this device is easily used by nonspecialist physicians. The application of a simple sensor sticker provides essential information for the management of a stroke patient. 

**Figure 5:**
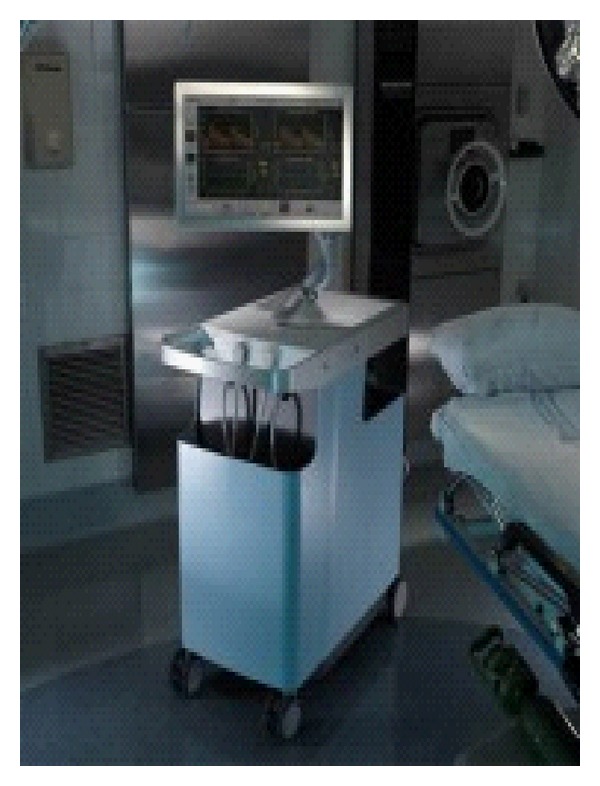
The “Presto” Cranial blood flow monitor. Courtesy of Physiosonics.

Another tool of significant importance for the neurosurgeon specialist in emergencies is the Presto, which monitors cranial blood flow without a sonographer (Figure 5). This is a device that has received FDA clearance and it allows any clinician to perform cranial blood flow monitoring without a professional sonographer. The system features proprietary autolocating technology that allows users to easily locate and keep the ultrasound beam focused on the cerebral artery [25]. The Presto 1000 Flow Monitor will help with ICU and surgical patients, by detecting abnormal changes in cerebral blood flow and alerting the attending nurse, bringing further institutional resources to diagnose the cause. These flow anomalies may be indicators of severe conditions, such as vasospasm from aneurysm ruptures or head trauma, disturbances in brain autoregulation or MCA blood clots [26]. Taking into account its function, and user-friendliness, this can be really considered as a vital device for neurosurgical emergencies. Without the specific knowledge of a sonographer, even a junior registrar can measure cranial blood flow and rather rapidly provide crucial information to the specialist. This ensures that appropriate treatment is rapidly provided and may even save the patient's life.

**Figure 6:**
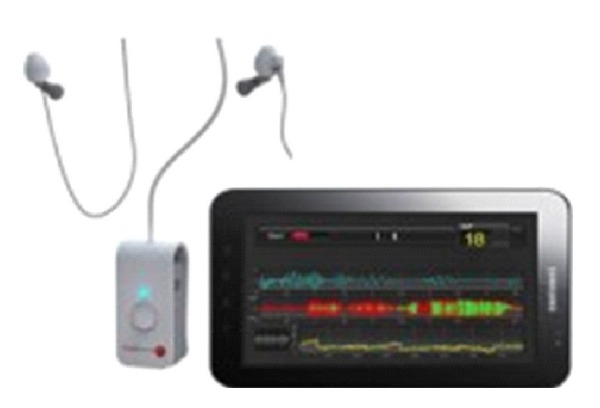
The “HeadSense” intracranial pressure monitor. Courtesy of HeadSense Company.

An essential device for emergencies in neurological surgery is the HeadSense intracranial pressure monitor (Figure 6). In this device, the ear buds may help avoid having to drill into the skull. Elevated transcranial pressure from an injury or disease is a dangerous condition that can be life-threatening, painful, and debilitating. Because our thick skulls do not allow traditional external pressure monitoring, invasive methods that penetrate the skull have been used. This is why intracranial pressure monitoring continues to be rooted in the 20th century. With simple ear buds, even a nonspecialist junior physician can easily decide whether the patient's intracranial pressure needs conservative or surgical treatment. This decision can be crucial for the patient's life, as well as for economic health management. HeadSense, a company from Netanya, Israel, has been working on a noninvasive monitor that sends and detects sound waves to measure intracranial pressure. It looks like a generic mp3 player but, instead of playing music, the ear bud plays tones of different frequencies and measures how they sound from the other side of the head. A Bluetooth-enabled tablet receives the data from the HeadSense device and analyses it to provide a final output of the pressure [27].

**Figure 7:**
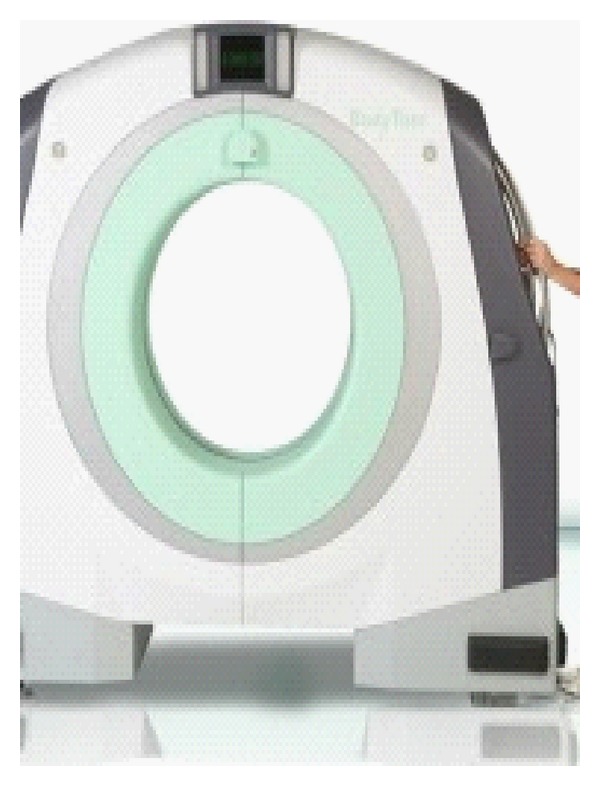
The “Bodytom” portable full body CT scanner. Courtesy of Neurologica.

Furthermore, the portable full body CT scanner—BodyTom—has been awarded the CE mark and is another important device for the emergency department (Figure 7). This is a battery powered tomograph and can be transported to the patient from room to room, allowing it to be used in places such as the clinic, the ICU, and the emergency department. The specifications of the scanner itself include a 32 slice CT with a 85 cm gantry and 60 cm view [28]. The BodyTom is the latest development in NeuroLogica's portable computed tomography imaging line. The battery powered BodyTom can be transported from room to room and is compatible with PACS, surgical navigation, electronic medical records, and planning systems. Its unique capabilities provide high quality CT images wherever needed, including the Clinic, ICU, operation room, and Emergency/Trauma Department. The combination of rapid scan time, flexible settings, and immediate image viewing makes the BodyTom a valuable tool to any facility needing versatile real-time CT imaging [29].

By providing real-time updates as the surgery unfolds, the scanner eliminates the need to move patients between operating room and radiology suite and makes surgery safer. BodyTom can obtain images of the entire spine in one pass, providing detailed three-dimensional images of both bone and soft tissue that is unavailable using the flat-panel fluoroscopic imaging that is typically found in most operating suites. This enables the surgeon to assess any developing complications before the patient leaves the operating room [30].

The importance of this device is (more than) obvious. You do not have to be a specialist to support its usefulness in emergencies. With this portable CT scanner, you can always have immediate neuromonitoring, even in the smallest medical centres. Moreover, the scanner is cost-effective, as it can be used by all the departments of the institute, especially if we are dealing with a small capacity medical centre.

**Figure 8:**
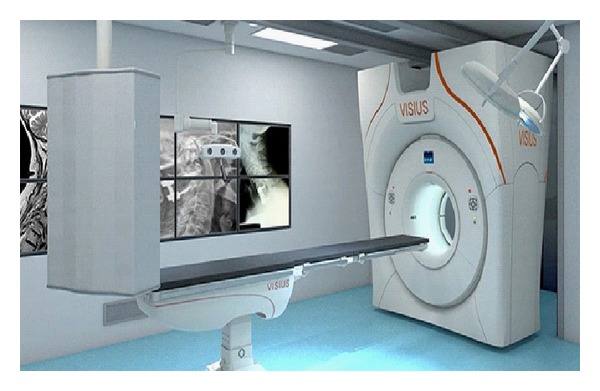
The ceiling-mounted intraoperative “VISIUS iCT”. Courtesy of IMRIS Company.

Remaining with the theme of diagnostic testing and computed tomography monitoring, the IMRIS CT (Figure 8) should be mentioned. IMRIS Inc. (IMRIS) designs, manufactures, and markets the VISIUS Surgical Theatre, a multifunctional surgical environment that provides intraoperative vision to clinicians. Designed to fulfil a hospital's clinical needs, the VISIUS Surgical Theatre can incorporate magnetic resonance (MR) imaging, computed tomography (CT) imaging, and X-ray angiography in a number of configurations, providing intraoperative images of diagnostic quality. IMRIS sells the VISIUS Surgical Theatres globally to hospitals that deliver clinical services to patients in the neurosurgical, cerebrovascular, and cardiovascular markets. The VISIUS Surgical Theatre incorporates magnetic resonance imaging, CT, and fluoroscopy into multipurpose surgical suites to provide truly intraoperative imaging for specific medical applications [31].

The FDA has given clearance to IMRIS for its ceiling-mounted intraoperative CT system, the VISIUS iCT. This is the world's first and only such system that rides on a ceiling-mounted track and can be deployed around the patient within 30 seconds when necessary. Because the system does not touch the ground, there are many fewer things to keep in mind when deploying the scanner, and the operation can be resumed quickly once the scan is done [27]. It is obviously an extraordinary device. It saves time for the treatment of the neurosurgical patient in the operation theatre of the emergency department. However, this is an extremely specialist-oriented machine and its use can only be focused in special medical centres. It cannot be considered as the frontier tool for neurosurgical emergencies, but may be regarded as an essential device in reference neurosurgical centres. State-of-the-art CT imaging is then accessed effortlessly in the operation room (OR).

Developed for cranial and spinal neurosurgery, VISIUS iCT is a specialized multifunctional surgical theatre that brings state-of-the-art image quality directly to patients in the OR. The first and only ceiling-mounted intraoperative CT travels on-demand to the patient—without introducing additional risks by moving the patient or having anything touch the floor—and preserves the OR protocols, including optimal surgical access and techniques [32]. 

VISIUS iCT is a state-of-the-art surgical theatre that provides personalized dose management together with diagnostic quality imaging during the surgical procedure, in order to assist surgeons in critical decision-making. The 64-slice scanner effortlessly moves into and out of the operating room during surgery, using ceiling-mounted rails to ease workflow. This enables multiple room configurations to meet both clinical requirements and increased utilization without compromising image quality or exam speed.

Patient transport and the need for the floor-mounted rails used in other systems are eliminated, opening up valuable OR space and allowing unimpeded movement of surgical equipment and simplified infection control. The system also offers the longest scanner travel range on the market today.

In addition, VISIUS iCT features a suite of software applications, such as 3D volume rendering, in order to aid surgical planning and dose reduction. This considers each patient's unique characteristics and needs to maximize image quality and minimize dose. “State-of-the-art dose management is one of the keys to driving the adoption of iCT to guide surgical procedures,” Graves said.

The system software allows healthcare practitioners to visualize dosage prior to scan and adjust settings based on the specific clinical need, with detailed dosage reports produced after each scan [33].

**Figure 9:**
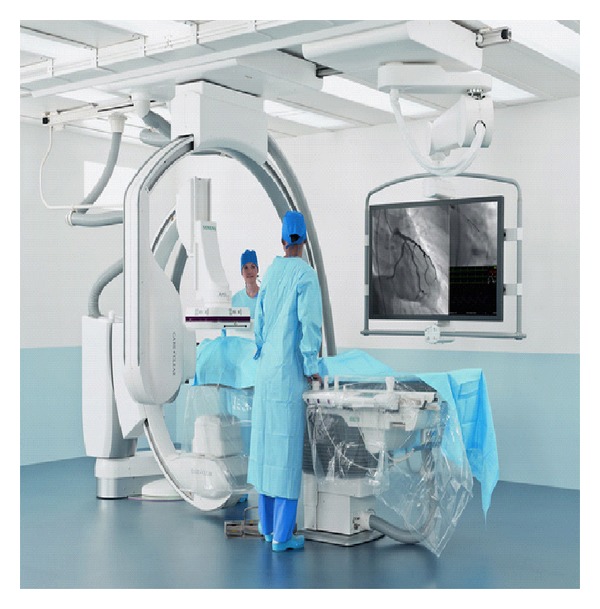
The Artis angiography system, courtesy of Siemens Company.

Finally, another new device that would be a great assistance to the specialist in the ED is the new angiography system from Siemens (Artis Q, Artis Q. zen), which has been awarded FDA approval (Figure 9). The device features the latest X-ray tubes and detectors, which together deliver the highest quality imaging and are capable of spotting tiny vessels in the brain and heart during interventional procedures. The system also features low radiation dose modes that allow imaging at lower quality when the best imaging is unnecessary [16]. This new angiography system though is also—as IMRIS CT—a device that cannot be used as frontline tool for neurosurgical emergencies. It must be used by specialists. Nevertheless, the features of the new angiography system make it an important implement for the structure of a developed neurosurgical centre, to improve the quality of patient treatment. The new imaging component techSiemens Artis Qnology is touted by Siemens as a “revolutionary new X-ray tube and detector technology” that will improve image quality and fine detail resolution, all with a dose level that is lower than the traditional tube and detector technology presently being used. The X-ray tube from Siemens now utilizes flat emitter technology rather than the traditional coiled filament technology. This is now coupled with the new detector design that utilizes crystalline silicon rather than amorphous silicon as the detector material. Siemens claims that the new design provides a more homogenous design with better signal amplification, while at the same time reducing the noise at ultralow dose settings. Thus, it provides better images at lower power and doses. 

Siemens claims that the new combination of tube and detector provides visibility of small vessels at a rate that is 70% higher than with the previous generation technology. If this is proven to be clinically valid in everyday use, the promise of better resolution at lower power and lower dose levels will be a significant step, as interventional procedures become more and more complex with longer times. This may translate into the potential for extended exposure to radiation during the procedure [34].

In addition to the hardware innovations, there are several new software applications. The Artis Q and Q. zen will be the first angiography systems to feature IVUS map, integrating intravascular ultrasound (IVUS) with angiographic images. With the simultaneous views of the vessels' interior wall via IVUS with precise location on the angioimage, IVUS map efficiently supports physicians in diagnostic testing and stent placement. CLEARstent Live enhances the visibility of stents in real time during therapy whilst simultaneously stabilising the image, resulting in a clear image of the intervention without time lag. Other new 3D applications, such as syngo DynaCT micro, provide substantial improvements in spatial resolution, by enhancing the smallest details in crucial areas such as imaging of intracranial stents or other miniscule structures, such as the cochlea in the inner ear. Organs such as the lungs can be imaged in 3D in less than three seconds with syngo DynaCT Highspeed, reducing the number of motion artefacts and the amount of contrast agent required. For oncological procedures, personalised therapy is the key to improve the access to the disease and to improve patient outcomes. A new 3D functional imaging protocol, syngo DynaPBV Body, shows blood distribution by means of colour coded cross-sectional blood volume maps, along with quantitative measurement of blood volume in lesions, in order to assess changes in perfusion over the course of treatment [35]. 

## 4. Ethics 

The last twenty years have been the rise of neuroscience, molecular biology, and brain imaging technologies, which have changed our understanding of the human brain. Neuroscientists are currently working on the development of an array of therapeutic cognitive enhancements for humans. These technologies are in the pioneering stage and promise further insights into our understanding of the brain. Some scientists consider that the twenty first century will be to neuroscience as the twentieth century was to physics [36]. 

The sheer size of the brain's neural network, comprising approximately 100 billion neurons and over 100 trillion synaptic connections, will represent an enormous challenge for NBIC in the future. Fantasies about mind control will remain elusive, since science has yet to determine how the brain encodes memory and more fundamentally how mind works [37]. Developments in BMIs, for example, will probably comprise two phases; the current phase consists of therapeutically based BMIs for disabled persons and in the second phase, BMIs will be developed to enhance cognitive and motor skills in healthy humans [37]. McGee and Maguire (2001) also predict a third phase in BMI development, which will involve the use of neural devices for information transfer capability [38]. While this phase is still a long way off, it does indicate future possibilities for BMI once the technology is developed. 

Episodes of neurosurgical care are associated with the expenditure of huge health system resources and have tremendous impact on quality of life and clinical outcomes, disproportionate to the size of our specialty and to the number of individual encounters. The complex medical, social, and ethical dimensions of these interventions preclude substituting mid-level providers, such as physician assistants and nurse practitioners, for the neurosurgeon. Many neurosurgical care episodes are concentrated at the extremes of age (i.e., at the beginning and end of life), focus on an unforgiving nervous system, and/or require continuous subspecialty coverage for stroke, hydrocephalus, and neurotrauma, and thus have tremendous impact on both healthcare and health system outcomes. For these reasons, neurosurgical practitioners have passionately embraced the modern era of medical education, training, and innovation as a method of improving the outcomes of our patients and for advancing our specialty. Neurosurgery has emerged as a leader and innovator, and is therefore an area of medical practice that should be targeted for additional support and enhanced attention to educational best practices, rather than cuts in economic support [39].

## 5. Discussion

ICT will no longer mean only intracranial tension. It will signify an even more important term—“Information and Communication Technology”. Distance today has become meaningless and geography has become history. Advances in medical technology and surgical techniques have dramatically improved diagnosis and treatment of most disorders, saving and extending lives. Yet the technology revolution has to bypass certain difficulties for urgent medical care in different hospital establishments. Tomorrow's neurosurgeon will be part of digital health, digital hospitals, EMR, HIS, telemedicine, telemonitoring, and mHealth. With a mini iPad or equivalent low cost tablet, he/she will be able to work with anyone, anytime, anywhere. Professor Google and Dr. Facebook will ensure that the patient is truly empowered, with real time access to almost the same exabytes of information as the neurosurgeon. “Caveat emptor”—let the buyer beware—the neurosurgeon of 2020 could very well be on the receiving end [40]. Ultimately, aligning preclinical and clinical standards on a large scale, as well as the vast use of modern technology healthcare tools in emergency neurosurgery, has the potential to greatly impact the lives of patients waiting for new treatments for brain and spinal cord injury in A & E Departments worldwide. In the 50 years since computed tomography was introduced to everyday medical use, the importance of new diagnostic technologies has become increasingly crucial. The pressure on physicians to be quick, effective, and to avoid any misjudgement of the patient has been transferred to our technology. Neurosurgery is obviously faced with many new opportunities and challenges, based on advanced technological approaches and molecular approaches to neurosurgical problems. Advances in technology have allowed the neurosurgeon to precisely locate abnormal tissue in the brain and spinal cord, thereby preserving normal tissues from surgical trauma. However, it is far from easy to predict the future role of the neurosurgeon. It is doubtful whether important turns and unexpected novelties can be foreseen by people of today, who are anchored to the palpable realities and submitted to all kinds of current limitations. Exposure to fantasies and wishful thinking will easily lead to errors and misunderstandings. Prophecies of this kind will be impregnated by current limitations. The reality is that a neurosurgeon will continually feel the force of the development and the need for new technology that will provide our patients with better outcomes and quality of life.
